# A DNA-conjugated small molecule catalyst enzyme mimic for site-selective ester hydrolysis†
†Electronic supplementary information (ESI) available. See DOI: 10.1039/c7sc04554a


**DOI:** 10.1039/c7sc04554a

**Published:** 2018-01-15

**Authors:** Moira L. Flanagan, A. Emilia Arguello, Drew E. Colman, Jiyeon Kim, Jesse N. Krejci, Shimu Liu, Yueyu Yao, Yu Zhang, David J. Gorin

**Affiliations:** a Smith College , Department of Chemistry , Northampton , MA 01063 , USA . Email: dgorin@smith.edu

## Abstract

The challenge of site-selectivity must be overcome in many chemical research contexts, including selective functionalization in complex natural products and labeling of one biomolecule in a living system. Synthetic catalysts incorporating molecular recognition domains can mimic naturally-occurring enzymes to direct a chemical reaction to a particular instance of a functional group. We propose that DNA-conjugated small molecule catalysts (DCats), prepared by tethering a small molecule catalyst to a DNA aptamer, are a promising class of reagents for site-selective transformations. Specifically, a DNA-imidazole conjugate able to increase the rate of ester hydrolysis in a target ester by >100-fold compared with equimolar untethered imidazole was developed. Other esters are unaffected. Furthermore, DCat-catalyzed hydrolysis follows enzyme-like kinetics and a stimuli-responsive variant of the DCat enables programmable “turn on” of the desired reaction.

## Abstract 2

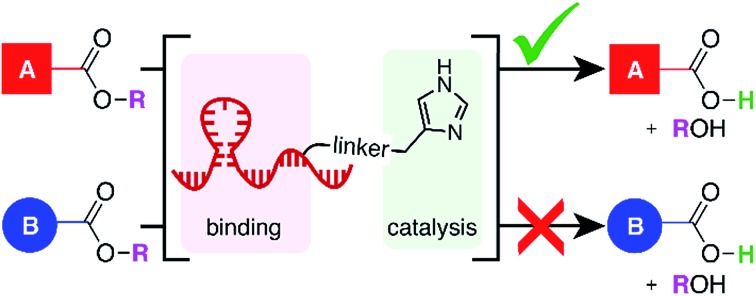
A DNA-imidazole conjugate, designed to mimic enzyme function, site-selectively hydrolyzes a target ester, but not other esters, with >100-fold rate enhancement compared to free imidazole.

## 

The challenge of “site-selectivity,” the transformation of one instance of a particular functional group among many, must be overcome in many chemical research contexts. Within a target molecule, site-selective reactions are needed for the synthesis of natural product derivatives or other functional small molecules, as illustrated in recent efforts toward site-selective alcohol functionalization in erythromycin^1^ (Fig. 1a) and C–H functionalization in substrates with multiple competing C–H bonds.^2^ Site-selectivity among molecules in a mixture is also crucial. For example, biomolecules contain many instances of the same functional group, such as amines on protein surfaces, making *in vivo* labeling difficult. Notably, the most successful strategies for biolabeling circumvent this challenge by incorporating a unique functional group, such as an azide, into the target molecule, which is available for highly chemoselective further reaction.^3^ While incredibly useful, such strategies require metabolic or genetic engineering to incorporate the unique tag, limiting the target scope. Site-selective chemical reagents that target a particular instance of a functional group are therefore highly desirable and would be useful in a wide array of applications in both chemical synthesis and chemical biology.

**Fig. 1:**
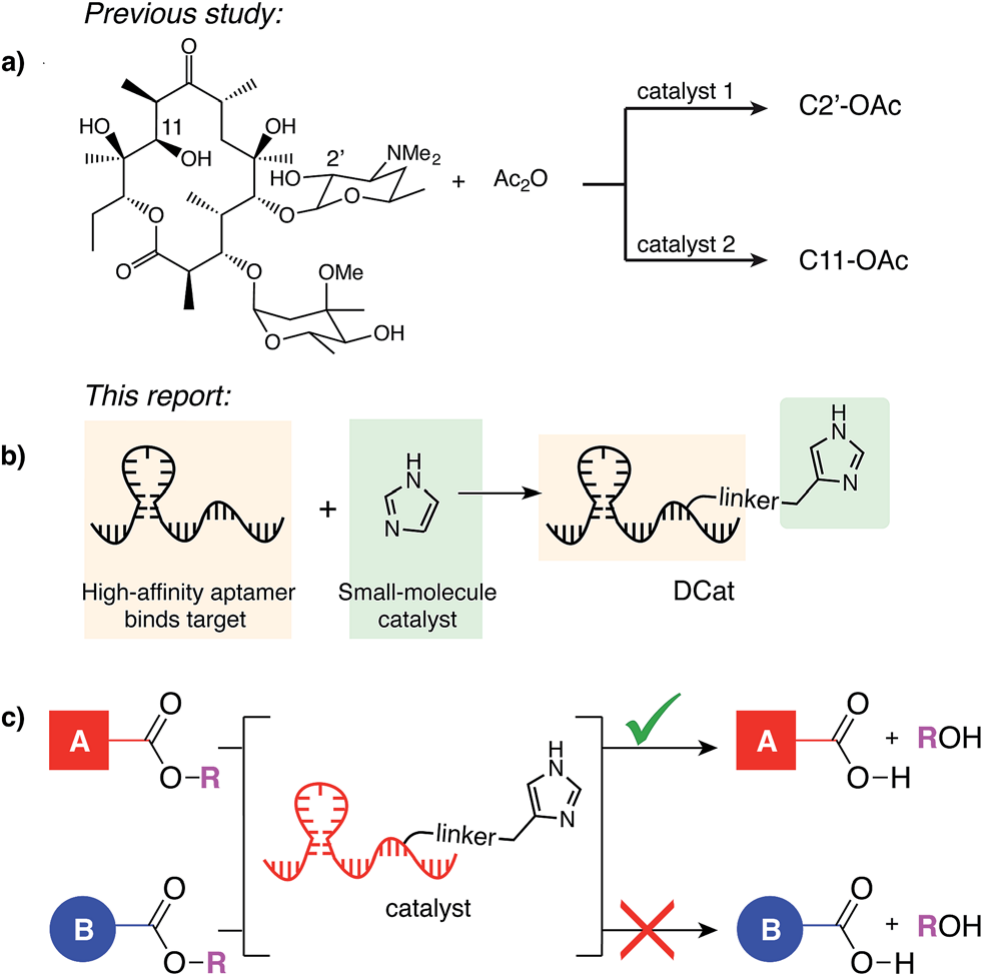
(a) Miller's catalyst-controlled, site-selective acetylation of erythromycin.^1^ (b) DCats assembled from small molecule catalyst (imidazole) and DNA aptamer. (c) DCat-catalyzed, site-selective ester hydrolysis.

When multiple instances of a functional group are present within a molecule or mixture, one site is often intrinsically more reactive, enabling substrate-controlled selective modification. As elegantly demonstrated by Miller,^1^ small molecule catalysts discovered by high-throughput screening can overcome intrinsic reactivity and direct a reaction to other sites. Many further developments in site-selective catalysis have drawn inspiration from nature.^4^ Protein enzymes recognize their substrates and catalyze reactions at a specific site; for example, proteases can hydrolyze one amide bond in the presence of many others,^5^ and restriction endonucleases sever the DNA backbone only at specific sequence locations.^6^ One factor contributing to enzyme selectivity is that binding to the target increases the effective concentration of the enzyme active site and the substrate, which in turn increases the rate of catalysis.

In analogy to biological enzymes, synthetic and semi-synthetic catalysts incorporating molecular recognition elements to promote substrate binding and accelerate a desired reaction have been investigated.^7^ Site-selectivity within vancomycin was demonstrated using peptide catalysts incorporating a vancomycin-binding domain7*a* and site-selectivity for protein labeling on live cell surfaces has been achieved using a reagent modularly assembled from a reactive small molecule catalyst and an antibody binding domain.^8^ Like proteins, nucleic acids may fold into three-dimensional structures that confer a specific function, with the additional advantages that they may be evolved to bind nearly any target *de novo*, synthesized easily and cheaply, and denatured reversibly.^9^ Given the promise and challenge of site-selective catalysis with peptide or protein recognition domains,^7^ we have initiated a research program to develop a class of catalysts that instead rely upon nucleic acid binding domains. Specifically, we aim to develop enzyme mimics by covalently linking selective, high-affinity DNA aptamers to versatile, efficient small molecule catalysts (Fig. 1b). The resulting DNA-conjugated small molecule catalysts (DCats) hold potential as a general class of site-selective reagents (Fig. 1c) for a wide range of reactions.

Nucleic acids have found some use as tools to control reaction selectivity in organic synthesis; well-developed examples include DNA-templated synthesis to control reactions of DNA-linked substrates^10^ and the use of DNA as a chiral ligand for enantioselective catalysis.^11^ The use of an RNA aptamer as a stoichiometric, non-covalent protecting group seminally demonstrated the potential of nucleic acids to change reaction site-selectivity within a non-DNA-linked substrate.^12^ Site-selective acylation of aminoglycoside antibiotics was achieved through blocking of some amines by the bound aptamer, which increased the relative reactivity of the remaining solvent-accessible amines.

Although limitedly studied, hybrid catalysts assembled from a nucleic acid recognition domain and a second reactive domain have demonstrated promising reactivity.^13^ In 2008, Marx and Hartig reported that proline linked to porphyrin-binding ssDNA effectively catalyzes the aldol reaction of a porphyrin-aldehyde.13*a* Although significant rate enhancement for the reaction of the porphyrin-aldehyde was observed with DNA-linked proline relative to free-proline, no studies with ostensibly non-binding aldehyde substrates were done. Very recently, Willner demonstrated that an aptamer-linked DNAzyme showed enhanced catalysis compared with the free DNAzyme.13b,^14^ Furthermore, the rate of reaction correlated with the binding affinity of the aptamer for its target, strongly suggesting that aptamers could be used as recognition domains in site-selective reagents and that selecting and/or designing binding and catalytic function separately can result in effective rate enhancement.^15^

Herein, we report a DNA-imidazole conjugate (**DCat1**) able to site-selectively increase the rate of ester hydrolysis in a cholic acid-derived ester (**1**); other esters not incorporating a cholate moiety are unaffected by the DCat.^16^ Ester hydrolysis was chosen for initial study due to its broad potential applicability, such as in the activation of caged probe molecules or pro-drugs,^17^ or in the functional perturbation of bioactive esters, such as *N*-acyl-homoserine lactone signals in quorum sensing.^18^ The DCat-catalyzed hydrolysis is dependent upon aptamer folding, a phenomenon which we exploited in the design and validation of a stimulus-responsive DCat that can be “turned on” by a pre-programmed signaling molecule. Analysis of hydrolysis kinetics reveals that **DCat1** is 100 times more effective per mole than free small molecule catalyst, and comparison of target and non-target esters illustrates that the DCat is highly site-selective.

## Results and discussion

### Design and synthesis of DCats for ester hydrolysis

**Fig. 2:**
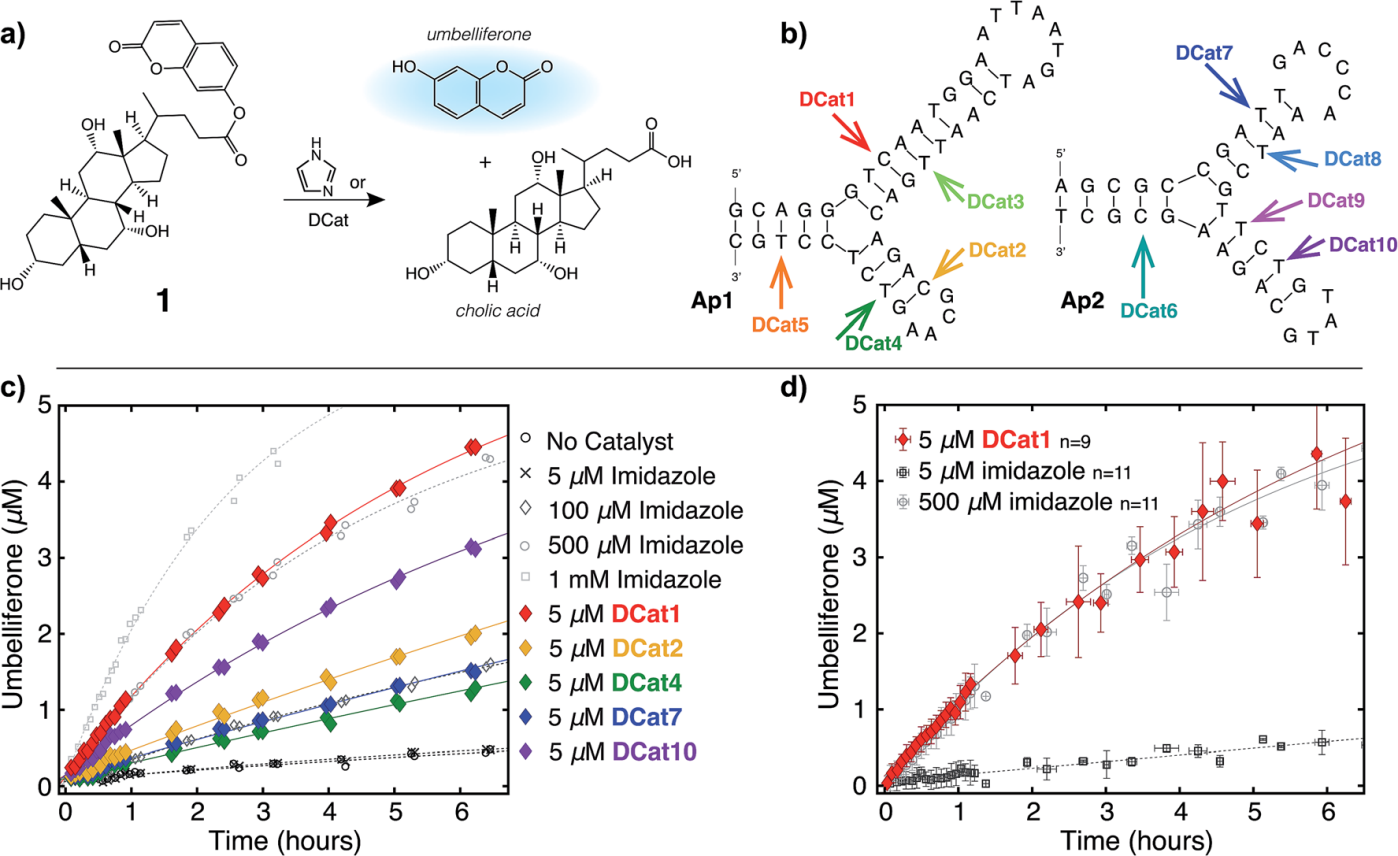
DCat enhances catalytic activity: (a) fluorogenic hydrolysis of **1** catalyzed by imidazole or a DCat. (b) Small DCat library constructed from two DNA sequences (**Ap1** and **Ap2**) with various imidazole attachment sites. Predicted secondary structures were found using Mfold.^19^ (c) Product formation over time from hydrolysis of **1** by each DCat (colored diamond markers). Control experiments with specified concentrations of free, untethered imidazole as shown (grey marker symbols). (d) Replicates and error bars for hydrolysis with **DCat1** and benchmark imidazole concentrations. Error bars indicate a standard deviation. See ESI† for further details.

In principle, either DNA or RNA aptamers may be incorporated into DCats. DNA has several technical advantages over RNA, including greater stability, and is therefore a more promising scaffold for interfacing with other reaction chemistries, even including transition metal catalysis.^10^,14b Cholic acid-derived umbelliferone ester **1** was chosen as the target for these proof of principle studies (Fig. 2a) since several aptamers that selectively bind cholic acid have been reported^20^ and the fluorogenic umbelliferone ester enables convenient determination of reaction progress.^21^ Imidazole was chosen as the hydrolytic small molecule catalyst due to its demonstrated compatibility with DNA.^16^

DCats were modularly assembled by conjugation of histamine to high-affinity, amine-modified cholic acid aptamers *via* the DSG crosslinker (see ESI, Fig. S1†). Two DNA sequences were used (**Ap1**, 48-mer, *K*_D_ = 5 μM and **Ap2**, 40-mer, *K*_D_ = 27.8 μM); both are proposed to bind cholic acid at a three-way junction site (as depicted in Fig. 2b).^20^ Since the optimal attachment point for the imidazole catalyst was uncertain, a small library of DCats was constructed from aptamers with different amine modification sites distributed around the proposed binding site (Fig. 2b).

### DCats show enhanced catalytic activity

To investigate the catalytic activity of each DCat, the initial rate of the DCat-catalyzed hydrolysis of **1** ([DCat] = 5 μM, [**1**] = 10 μM) was compared with the initial rate of the same reaction catalyzed by free imidazole ([imidazole] = 5 μM). Since each DCat molecule contains one imidazole moiety, this experiment illustrates whether the DNA influences the rate of imidazole-catalyzed hydrolysis; if no rate enhancement is conferred by the DNA domain, then the reaction rate should match that observed with free imidazole. For additional comparisons, hydrolysis of **1** with no catalyst and increasing concentrations of free imidazole (100 μM, 500 μM and 1 mM) was also performed (Fig. S4†). Fluorescence from liberated umbelliferone, a reaction product, was monitored over time (Fig. 2c).

Strikingly, several DCats in the library, including the five shown in Fig. 2c, showed significant rate enhancement compared with equimolar imidazole. The reactions with imidazole at varying concentrations provide a further benchmark to evaluate the relative rates of hydrolysis. Several DCats (**DCat2**, **DCat4** and **DCat7**) at 5 μM concentration catalyze ester hydrolysis at initial rates comparable with 100 μM (20-fold excess) free imidazole, while the rate with **DCat10** is even faster. However, the most effective construct, **DCat1**, hydrolyzes **1** at an initial rate comparable to 500 μM (100-fold excess) untethered imidazole (Fig. 2c and d).

To confirm this qualitative assessment, the effective first order rate constants were determined for each reaction by dividing the slope of the linear region at early times by the concentration of substrate (*k*_obs_, Table 1). The pseudo first-order rate constants for 5 μM **DCat1** and equimolar imidazole differ by an order of magnitude (*k*_obs_ = 0.09 h^–1^*vs.* 0.009 h^–1^). However, 5 μM of imidazole does not accelerate hydrolysis significantly above the background (uncatalyzed) rate, so a more informative comparison is 5 μM **DCat1***vs.* an equally effective concentration of imidazole. Notably, the rate constant for 500 μM imidazole is identical to that for 5 μM **DCat1** (0.11 h^–1^*vs.* 0.09 h^–1^). That is, **DCat1** is as effective as a 100-fold excess of free small molecule catalyst. This becomes more apparent when the second-order rate constants are extrapolated from the effective first order rates.^22^ Both 5 and 500 μM imidazole yield second order rate constants ∼0.2 mM^–1^ h^–1^, while *k*_app_ for **DCat1** is two orders of magnitude larger (*k*_app_ = 17 mM^–1^ h^–1^).^23^ Taken together, these experiments show that linking the DNA aptamer to imidazole dramatically increases the rate of ester hydrolysis of **1** compared with free imidazole, and that this rate enhancement varies with the site of DNA modification.

**Table 1:** Effective first-order kinetic constants for **DCat1** and free imidazole. **DCat1** (*n* = 9), imidazole (*n* = 11). Error is defined as one standard deviation

Catalyst	*k*_obs_ ^*a*^ (h^–1^)	*k*_app_ ^*b*^ (mM^–1^ h^–1^)
5 μM **DCat1**	0.09 ± 0.03	17 ± 5
500 μM imidazole	0.11 ± 0.04	0.21 ± 0.07
5 μM imidazole	0.009 ± 0.003	0.18 ± 0.07

^*a*^Observed pseudo first-order rate constant determined from the initial reaction rate: *k*_obs_ = *v*_i_/[*S*_0_] = *v*_i_/10 μM.

^*b*^Apparent, extrapolated second-order rate constant determined by: *k*_app_ = (*k*_obs_ – *k*_back_)/[catalyst] where *k*_back_ = 0.008 ± 0.002 h^–1^, the uncatalyzed hydrolysis rate.

### DCat catalysis is dependent on DNA tertiary structure

**Fig. 3:**
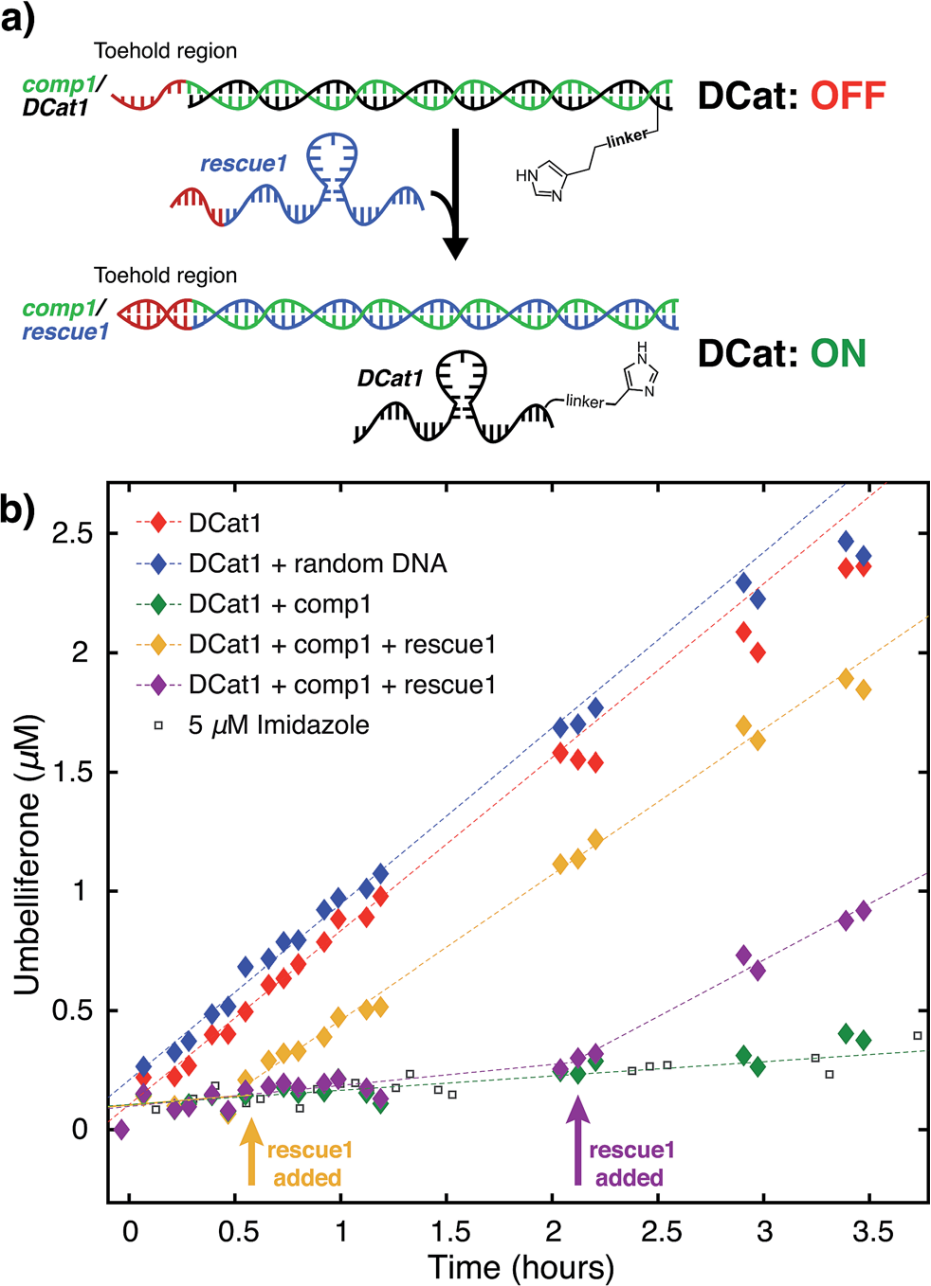
DCat “turn-on” using toehold displacement: (a) inactive **DCat1** rescued by displacement of **comp1** with **rescue1**. (b) Product formation over time from hydrolysis of **1** by **DCat1** in the presence (green diamond) and absence of **comp1**. DCat activity “turns on” at 35 minutes (yellow diamonds) or 130 minutes (purple diamonds) upon addition of **rescue1**.

We hypothesized that the enhanced rate of ester hydrolysis observed upon tethering imidazole to the DNA aptamer is due to binding of the aptamer to the cholate moiety in **1**, which increases the effective concentration of imidazole and **1**.^10^ Because aptamer function is known to depend on the ssDNA folding into the active three-dimensional structure, this suggests that DCat activity requires aptamer folding. To test this, DCat-catalyzed hydrolysis of **1** was conducted in the presence of an ssDNA complementary to the aptamer (**comp1**, Fig. 3).^24^ The addition of equimolar complementary DNA ([**comp1**] = 5 μM) to the standard reaction conditions ([DCat] = 5 μM, [**1**] = 10 μM) resulted in complete loss of **DCat1** activity—the hydrolysis of **1** proceeded identically to the reaction with 5 μM free imidazole (which is also indistinguishable from the reaction run without any added catalyst). A control experiment demonstrated that addition of a non-complementary ssDNA (**random DNA**) had no effect on the rate of hydrolysis; the DCat-mediated reaction proceeded rapidly just as in the case where no additional DNA was added.^25^ These results indicate that correctly-folded aptamer is necessary for catalysis and strongly suggests that the aptamer domain of **DCat1** binds to **1** during the catalytic cycle.

The “turn-off” response of **DCat1** to the presence of **comp1** suggests that DCats have the potential to be stimuli-responsive, dynamic reagents that can be controllably and reversibly deactivated and activated. To demonstrate this, a system for controllable, time-dependent DCat activation was developed using toehold displacement (Fig. 3a).^26^ In this assay, the DCat is initially incubated with **comp1** before the addition of substrate (Fig. 3a). Hybridization inhibits DCat activity, as described above. The hybridized complementary strand contains an extra toehold region of 10 nucleotides which provides an opportunity for strand displacement. When **rescue1**, ssDNA with a primary sequence that is fully complementary to **comp1**, is added, it will displace **DCat1**, which should rescue DCat function and “turn on” the hydrolysis of **1**. Delightfully, after incubation of DCat with **comp1**, DCat-catalyzed hydrolysis at enhanced rates was observed only upon addition of the **rescue1** strand (Fig. 3b). This was demonstrated at two different time points (0.55 hours and 2.1 hours) to illustrate temporal stimulus-responsive control of DCat activity. This type of behavior is highly desirable for a variety of applications in DNA nanotechnology.^27^ A DCat could be easily incorporated into other nucleic acid based-architectures, including hydrogels, DNA origami, and other nanostructures, expanding the possible applications of DCats, and offering an avenue for incorporating small molecule catalysts into DNA architecture and nanotechnology.

### DCat shows site-selective rate enhancement

**Fig. 4:**
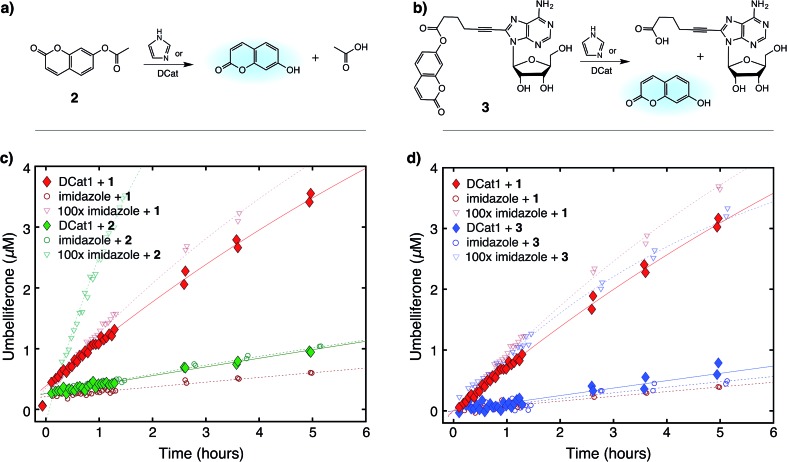
Site-selective ester hydrolysis (a) fluorogenic hydrolysis of **2**. (b) Fluorogenic hydrolysis of **3**. (c) **DCat1**-catalyzed reaction of **1** (red-filled diamonds) *vs.***2** (green-filled diamonds). Reactions with benchmark imidazole concentrations also shown (open shapes). (d) **DCat1**-catalyzed reaction of **1** (red-filled diamonds) *vs.***3** (blue-filled diamonds). Reactions with benchmark imidazole concentrations also shown (open shapes). See ESI† for further details.

Next, the site-selectivity of **DCat1** was investigated by comparing the rate of DCat-mediated hydrolysis of **1** with the rates of other umbelliferone esters. The results presented thus far suggest that target recognition and binding is essential for the enhanced rate of DCat-catalyzed hydrolysis of **1**, and therefore other esters not recognized by the DCat's DNA aptamer domain should not be affected. To test this hypothesis, the **DCat1**-catalyzed hydrolysis of esters **2** and **3**, derived from acetic acid and adenosine, respectively, were investigated (Fig. 4a and b). For all three esters (**1**, **2**, **3**), uncatalyzed background hydrolysis is indistinguishable from reactions containing 5 μM imidazole (see Table 2 and ESI†) so enhancement above 5 μM imidazole signifies meaningful rate increase. Notably, no rate enhancement from the DCat is observed in reactions with **2** or **3** (Table 2); the rates of hydrolysis are very similar whether the esters are treated with 5 μM **DCat1** or 5 μM free imidazole (Fig. 4c and d). In contrast, the rate of hydrolysis of **1** is clearly enhanced in the presence of **DCat1**, which illustrates site-selectivity for **1**, and not **2** or **3**.

**Table 2:** Rate Enhancement calculated for each ester with **DCat1** and imidazole. Error is defined as one standard deviation

Catalyst	Rate enhancement: *k*_obs_/*k*_back_^*a*^		
	Substrate **1**	Substrate **2**	Substrate **3**
5 μM **DCat1**	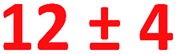	1.2 ± 0.5	0.9 ± 0.5
500 μM imidazole	15 ± 5	16 ± 3	8 ± 3
5 μM imidazole	1.1 ± 0.3	1.1 ± 0.5	1.1 ± 0.5

^*a*^Rate enhancement is the ratio of the pseudo first-order rate constant of a catalyzed reaction, *k*_obs_, divided by *k*_back_ where *k*_back_ is the self-hydrolysis rate in the presence of no catalyst. For 10 μM **1**: *k*_back_ = 0.008 ± 0.002 h^–1^; for **2**: *k*_back_ = 0.013 ± 0.008 h^–1^; for **3**: *k*_back_ = 0.011 ± 0.008 h^–1^.

To quantify these observations, a rate enhancement was determined for each catalyst–ester pair by taking the observed first-order rate constant and dividing by the background reaction, (*k*_obs_/*k*_back_). At the lowest concentration of 5 μM, imidazole has *k*_obs_/*k*_back_ of ∼1 regardless of the ester, corresponding to a lack of rate enhancement. Imidazole at higher concentration gives rate enhancement across all three substrates; no appreciable site selectivity is observed. In contrast, the rate enhancements for **DCat1** are negligible for hydrolysis of the non-target esters **2** and **3** (*k*_obs_/*k*_back_ = 1.2 and 0.9 respectively), but significant for hydrolysis of **1** (*k*_obs_/*k*_back_ = 12). This demonstrates a high degree of site-selectivity, as all three molecules contain umbelliferone esters, but only one undergoes rapid hydrolysis. Excitingly, this highlights the potential use of DCats as site-selective reagents in mixtures.

### Kinetic analysis

**Fig. 5:**
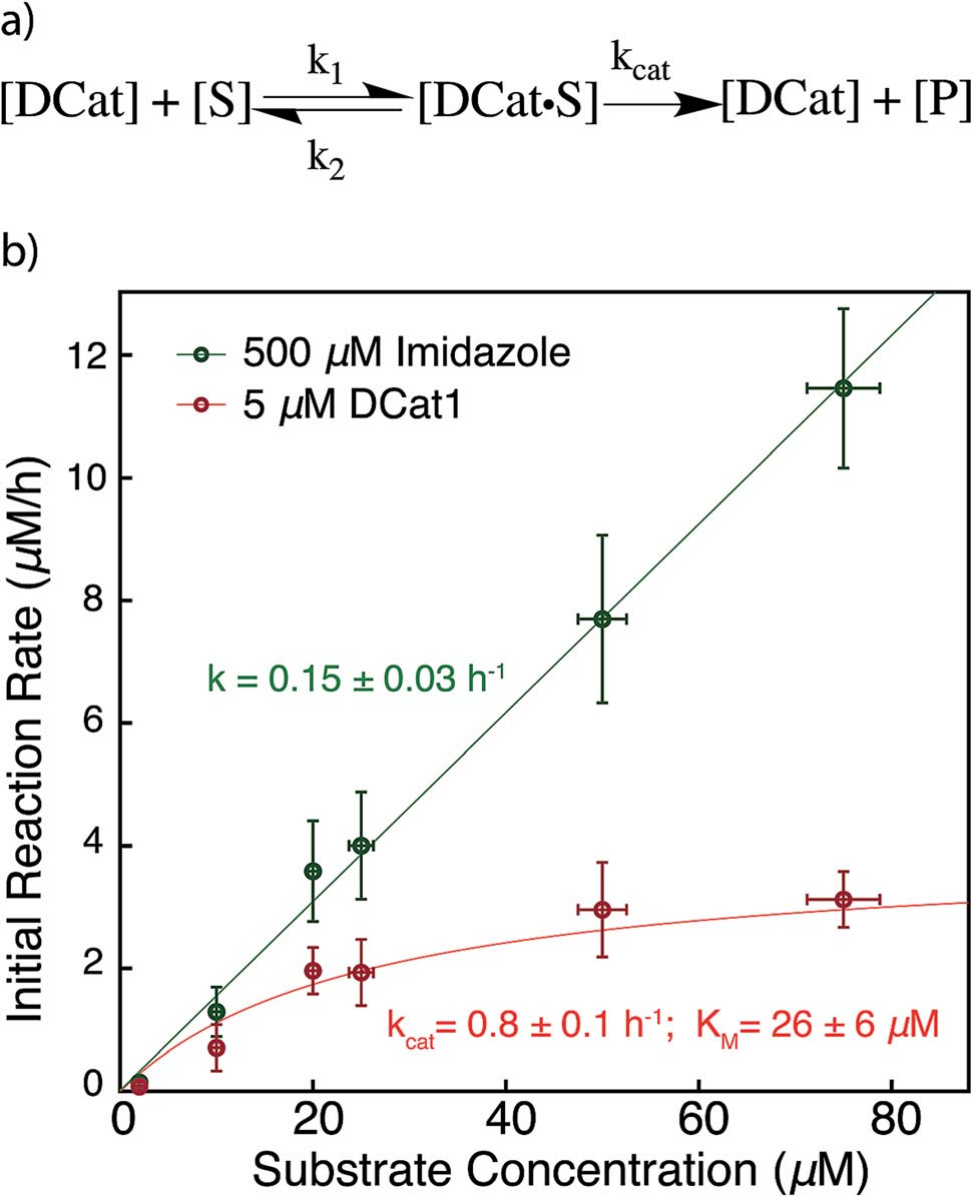
Proposed enzyme-like mechanism for DCat catalysis (a) Michaelis–Menten model (b) dependence of initial reaction rate on substrate concentration for DCat-catalysis (red) with Michaelis–Menten parameters *vs.* imidazole (green) catalysis with pseudo first-order rate constant. Error bars ± 1 S.D. See ESI† for further details.

The results above are broadly consistent with non-covalent binding of DCat to the substrate as a key step in the catalytic cycle. This suggests an analogy to protein enzymes, where formation of an enzyme–substrate complex prior to covalent transformation is generally proposed.^28^ If the reactions proceed within an overall mechanistic framework closely resembling natural protein enzymes, DCats may be considered as modularly assembled synthetic enzymes, and the reaction kinetics with DCats should closely resemble enzyme kinetics (Fig. 5a). To test this, the **DCat1**-catalyzed ester hydrolysis was investigated over a range of substrate concentrations ([**1**] = 10–75 μM *etc.*), initial rate constants were determined (Fig. S5†), and the results were fit to the Michaelis–Menten equation (Fig. 5). From this, *k*_cat_ (0.8 h^–1^) and *K*_M_ (26 μM) were determined. The *K*_M_ value is consistent with the reported *K*_D_ of the cholic acid aptamer.^20^ The *k*_cat_ value is small compared with natural enzymes,^29^ but higher than many DNAzymes14*b* and *de novo* designed enzymes.13*b* Furthermore, kinetic comparison of **DCat1** with free imidazole highlights significant rate advantages for the DCat.

To better compare the mechanisms of the DCat-catalyzed hydrolysis and the reaction catalyzed by free imidazole, the imidazole-catalyzed reaction ([imidazole] = 500 μM) was also investigated at multiple substrate concentrations (Fig. 5). The imidazole-catalyzed reaction exhibited second-order kinetics (see ESI†), so each single run fits a simple pseudo first-order kinetic model.14b,d As expected for a typical second-order kinetic reaction, the initial rate increases linearly as [**1**] increases. The pseudo first-order rate constant (*k*_obs_) is 0.15 h^–1^ and no saturation kinetics are observed. Comparison of the reaction rates at high substrate concentrations clearly illustrates that the DCat kinetic model (which shows saturation) is completely different from free imidazole, despite the fact that an imidazole moiety is crucial to reaction progress in both cases.

At low substrate concentration, the “first-order region” of Michaelis–Menten kinetics, both the DCat and imidazole catalyzed reactions may be considered pseudo first-order reactions and thereby meaningfully compared.^30^ The 500 μM imidazole-catalyzed reaction follows a pseudo first-order *k*′_obs_ = 0.15 h^–1^, while catalysis with 5 μM **DCat1** gives *k*′_obs_ = *k*_cat_[*E*_0_]/*K*_M_ = 0.15 h^–1^. The identical *k*_obs_ obtained for each reaction are consistent with the observations in Fig. 2; the rate of product formation is the same. Additionally, the effective first-order rate constant for **DCat1** from Table 1 also agrees with the first-order region of this Michaelis–Menten description. When the second order rate constants are extrapolated for each catalyst, the rate constant for imidazole is found to be *k*′_app_ ≈ 0.3 mM^–1^ h^–1^ while **DCat1** gives *k*′_app_ ≈ 30 mM^–1^ h^–1^ (Table 3). That is, the intrinsic rate for **DCat1**-catalyzed hydrolysis of **1** is two orders of magnitude higher than that of free imidazole. These results are very encouraging for the potential application of DCats to biolabeling. In biological mixtures, the desired target molecule is often low (0.1–50 μM range for proteins in the cytoplasm)^31^ so the rate enhancement conferred by a DCat at low substrate concentrations (in the Michaelis–Menten “first order region”) is highly relevant and promising.

**Table 3:** Michaelis–Menten Analysis of **DCat1** Compared with imidazole. The Michaelis–Menten parameters are used to compare 5 μM **DCat1** (*n* = 4) to 500 μM imidazole (*n* = 6) kinetic behavior over increasing substrate concentration. Error is defined by one standard deviation

Catalyst	*k*_cat_ (h^–1^)	*K*_M_ (μM)	*k*_app_ ^*a*^ (mM^–1^ h^–1^)	*k*′_app_/*k*′_app,imid_
500 μM imidazole	—	—	0.30 ± 0.07	1
5 μM **DCat1**	0.8 ± 0.1	26 ± 6	31 ± 8	103

^*a*^Extrapolated apparent second-order rate constant determined by *k*′_app_ = *k*_obs_/[catalyst].

## Conclusions

Synthetic catalysts that incorporate molecular recognition domains offer a strategy for achieving the site-selective transformation of one instance of a functional group. In this work, we demonstrated that a DCat, prepared by tethering imidazole to a DNA aptamer, increases the rate of ester hydrolysis of a specific target ester by >100-fold compared with equimolar untethered imidazole. No rate enhancement was observed with other esters, demonstrating site-selective catalysis. DCat-catalyzed hydrolysis followed enzyme-like kinetics at biologically relevant concentrations, and a stimuli-responsive, programmable variant of the DCat system exploiting toehold displacement was demonstrated. The fact that a DCat can be temporally controlled in this manner indicates that the system may be useful in drug-release and related applications.

In analogy to natural protein enzymes that both recognize their targets and catalyze specific chemical reactions, we have assembled an enzyme mimic that uses a DNA aptamer as a recognition domain while exploiting the efficiency and versatility of a small molecule catalyst. The use of functional nucleic acids as the binding domain promises that DCats can be prepared for a wide range of targets and applications, since SELEX enables the discovery of aptamers that bind nearly any target. Furthermore, both the aptamer and catalyst can be modularly substituted as necessary for a particular application or reaction chemistry. We envision that DCats may be useful in a broad array of applications, including in target-oriented synthesis to differentiate two instances of the same functional group within a single complex molecule and in bioconjugation efforts to target a particular protein or metabolite in a complex biological mixture. Experimental efforts to expand the scope of reactions mediated by DCats and to demonstrate selectivity in complex biological mixtures and other applications are currently underway.

## Conflicts of interest

There are no conflicts of interest to declare.

## Supplementary Material

Supplementary information
