# Supplementary material for: A DNA-conjugated small molecule catalyst enzyme mimic for site-selective ester hydrolysis
Source: Chem Sci. 2018 Jan 15;9(8):2105–12. doi: 10.1039/c7sc04554a (PMC5911826; doi:10.1039/c7sc04554a)
Supplement: Supplementary file 1 [file SC-009-C7SC04554A-s001.pdf]

Electronic Supplementary Material (ESI) for Chemical Science.
This journal is © The Royal Society of Chemistry 2018

Electronic Supporting Information for

A DNA-conjugated small molecule catalyst enzyme mimic for site-selective ester hydrolysis

By Moira L. Flanagan, A. Emilia Arguello, Drew E. Colman, Jiyeon Kim, Jesse N. Krejci, Shimu Liu, Yueyu Yao, Yu Zhang, David J. Gorin*
Smith College, Department of Chemistry, Northampton MA 01063
*dgorin@smith.edu

Table of Contents

Materials and Methods S3
General Information S3
Synthesis of DCats S3
Fluorogenic Assays for Ester Hydrolysis S6
Quantification of [umbelliferone] S6
Control Experiments: DCat structure S6
Data Analysis S8
Michaelis-Menten Kinetic Analysis S8
First-order Rate Law Parameters S8
*Kinetics of imidazole-catalyzed hydrolysis of substrate **1*** S10
Uncatalyzed Hydrolysis Rates S10
Toehold-Switch Experiments: S11
Synthesis of esters S13
Synthesis of substrate 1. . . . . S13
Synthesis of substrate 2. . . . . S13
Two-step synthesis of substrate 3 . . . . . S14
NMR data S16
Supplemental References S20

Materials and Methods:

General Information:

Unless otherwise noted, all reagents were obtained commercially and used without further purification. RP-HPLC purification was performed with Acetonitrile/0.1 M TEAA as the mobile phase at a rate of 1 mL/minute at 40 °C using a reverse-phase Zorbax Eclipse XDB-C18 column (5 µm, 4.6 x 250 mm). NMR spectra were referenced to chloroform or methanol and obtained with a Bruker 300 MHz NMR or Bruker 500 MHz NMR, as noted. DCat and DNA-construct mass were confirmed by ESI-MS using a Thermo Finnigan LGQ Deca XP Max mass spectrometer, while high-resolution mass spectral data to confirm mass of substrates **1** and **3** was obtained by the University of Massachusetts Mass Spectrometry Center.

Synthesis of DCats:

Cholic acid-binding DCats were synthesized from published aptamers.[1,2] Oligomers with C_6-NH_2 modifications at the desired base locations (Fig S1b) for each DCat were ordered from TriLink BioTechnologies (Table S1).

For each DCat synthesis, stocks of 20 mM disuccinimidyl glutarate (DSG, 5.2 mg, 16 µmol in 800 µL DMSO) and 30 mM histamine (2.8 mg, 24.8 µmol in 800 µL DMSO) were prepared. 500 µL of each stock were combined (new concentrations = 10 mM DSG and 15 mM histamine) and agitated for 10 minutes. A second solution containing DNA (20 nmol, 20 µL from 1mM stock in PBS buffer) and 40 µL 0.5 M TEA/HCl pH10 was prepared, and 240 µL of the DSG/histamine solution was then added to DNA solution. The reaction was vortexed for 30 minutes and incubated at room temperature overnight. Elution through an Illustra NAP-5 column (GE Healthcare) and RP-HPLC provided pure DCat. Concentration was determined with a Nanodrop 8000 spectrometer, and mass was confirmed by ESI-MS (Table S2).

a)

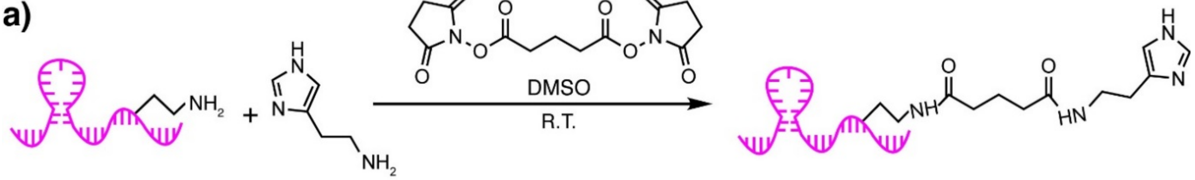

Figure S1: Scheme for synthesis of DCats

Construct	Aptamer Sequence
DCat1	5’ GCA GGG T**(dC-C6-NH$_2$)**A ATG GAA TTA ATG ATC AAT TGA CAG ACG CAA GTC TCC TGC 3’
DCat2	5’ GCA GGG TCA ATG GAA TTA ATG ATC AAT TGA CAG A**(dC-C6-NH$_2$)**G CAA GTC TCC TGC 3’
DCat3	5’ GCA GGG TCA ATG GAA TTA ATG ATC AAT **(dT-C6-NH$_2$)**GA CAG ACG CAA GTC TCC TGC 3’
DCat4	5’ GCA GGG TCA ATG GAA TTA ATG ATC AAT TGA CAG ACG CAA G**(dT-C6-NH$_2$)**C TCC TGC 3’
DCat5	5’ GCA GGG TCA ATG GAA TTA ATG ATC AAT TGA CAG ACG CAA GTC TCC **(dT-C6-NH$_2$)**GC 3’
DCat6	5’ AGC GCC GAT TGA CCC AAA TCG TTC TGT ATG CAG AAG **(dC-C6-NH$_2$)**GC T 3’
DCat7	5’ AGC GCC GA**(dT-C6-NH$_2$)** TGA CCC AAA TCG TTC TGT ATG CAG AAG CGC T 3’
DCat8	5’ AGC GCC GAT TGA CCC AAA **(dT-C6-NH$_2$)**CG TTC TGT ATG CAG AAG CGC T 3’
DCat9	5’ AGC GCC GAT TGA CCC AAA TCG T**(dT-C6-NH$_2$)**C TGT ATG CAG AAG CGC T 3’
DCat10	5’ AGC GCC GAT TGA CCC AAA TCG TTC **(dT-C6-NH$_2$)**GT ATG CAG AAG CGC T 3’

Table S1: Sequences of aptamers incorporated into the designated DCat. Amine-modified bases are in bold.

Compound	Ion Charge (Z)	Calculated *m/z*	Observed *m/z*
DCat1	-16	949.18	949.27
	-17	893.29	893.40
	-18	843.60	843.80
DCat2	-16	949.18	949.27
	-17	893.29	893.33
	-18	843.60	843.73
DCat3	-16	948.24	948.10
	-17	892.40	892.21
	-18	842.77	842.65
DCat4	-16	948.24	948.33
	-17	892.40	893.13
	-18	842.77	843.07
DCat5	-13	971.63	971.48
	-14	902.16	901.94
	-15	841.95	841.81
DCat6	-13	971.63	971.48
	-14	902.16	901.94
	-15	841.95	841.74
DCat7	-13	972.79	972.56
	-14	903.23	903.02
	-15	842.95	842.68
DCat8	-13	971.63	972.53
	-14	902.16	902.80

	-15	841.95	842.60
DCat9	-13	971.63	972.47
	-14	902.16	903.07
	-15	841.95	843.07
DCat10	-13	971.63	971.73
	-14	902.16	902.33
	-15	841.95	842.20

Table S2: MS characterization of DCat constructs

Fluorogenic Assays for ester hydrolysis:

Unless otherwise noted, reactions were performed in 40 µL volumes with 5 µM DCat and 10 µM substrate compound in buffer (20 mM Tris-HCl pH 7.4, 100 µM NaCl, 5 mM KCl, 2 mM $MgCl_2$, 1 mM $CaCl_2$) and 10% (v/v) DMSO. Before the addition of substrate, reaction mixtures were heated for 10 minutes at 95°C and cooled to 37°C at a rate of 0.1°C/s in a MJ Research PTC-2000 Peltier Thermal Cycler.[3] Hydrolysis reactions were run at 37°C. All experiments were repeated at least twice. To avoid confusion and clutter, graphs with many experimental conditions show only one representative experiment for each set of conditions.

Quantification of [umbelliferone]

Umbelliferone fluorescence was monitored (λ_{ex}=320 nm and λ_{em}=460 nm) for 36-120 hours after the addition of substrate to the reaction with a Molecular Devices SpectraMax i3x Multi-Mode Detection Platform. Umbelliferone concentration is linearly proportional to fluorescence, (Fig S2) enabling conversation of fluorescence units to product concentration. A standard ladder of umbelliferone solutions was included for every experiment for standardization.

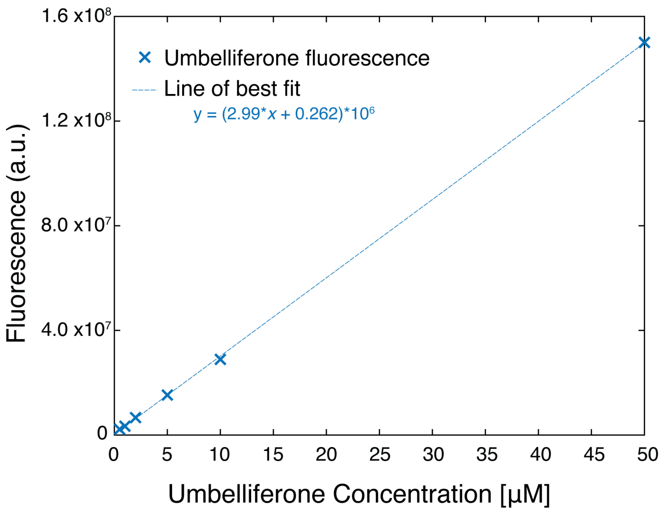

Figure S2: Umbelliferone fluorescence is linear with concentration shown in a representative calibration curve.

Control experiments: DCat structure

To ensure that the entire DCat construct is necessary for catalytic activity, we compared **DCat1**-catalyzed hydrolysis of **1** to control reactions where the DCat is replaced with a known starting material or side product from the DCat synthesis (Fig. S3). Additionally, a hydrolysis reaction was run in the presence of

both amine-modified Ap1 and free imidazole (Fig S4). No rate enhancement was observed, confirming that the covalent link between Ap1 and imidazole is essential. Catalytic activity is only observed with **DCat1**.

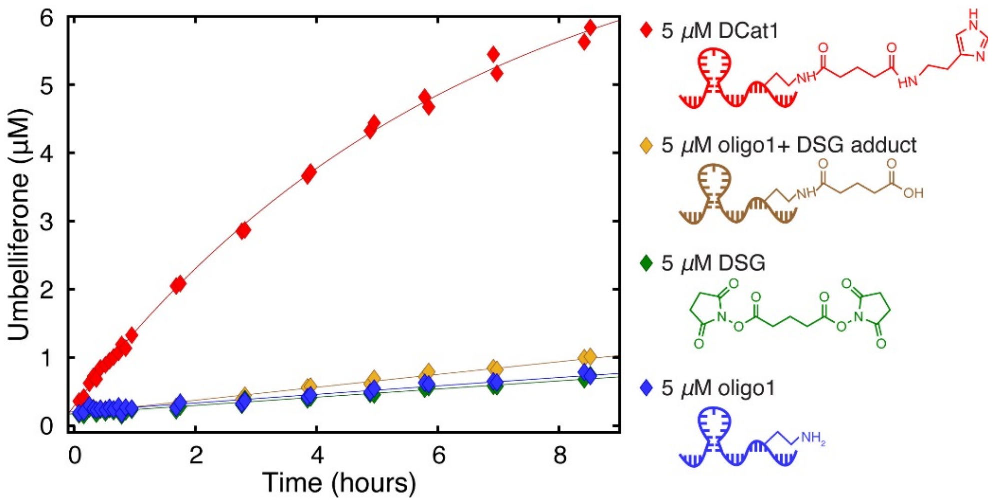

Figure S3: Control experiments for starting materials and side-products that might be present following the synthesis of **DCat1**. Reactions run in standard conditions.

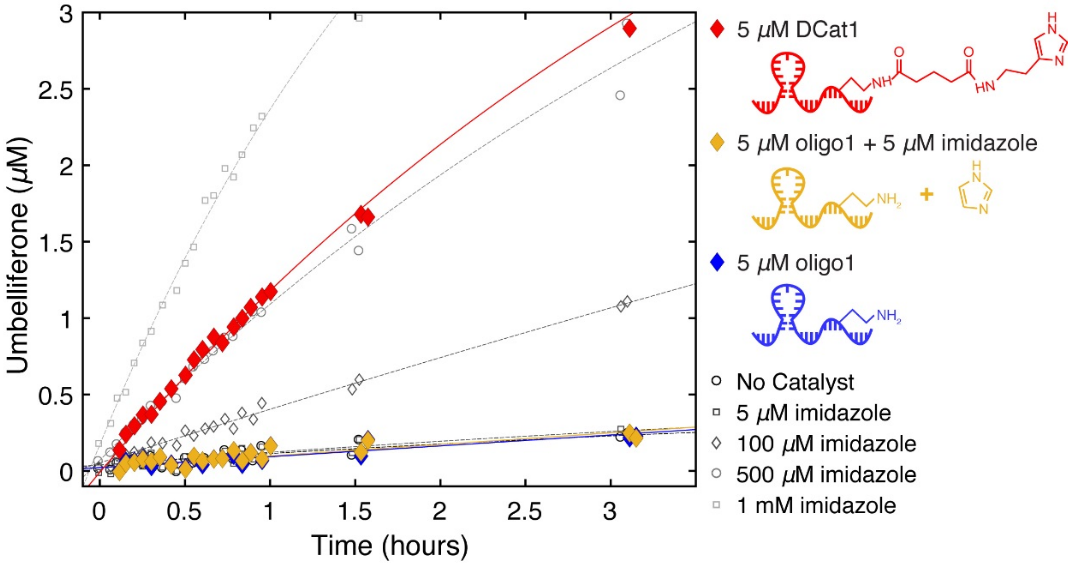

Figure S4: Control experiments of uncorrelated and untethered DNA and imidazole. Reactions run in standard conditions.

Data Analysis:

Michaelis-Menten Kinetic Analysis:

The Michaelis-Menten parameters (Fig 5) were determined by fitting the 5 uM **DCat1** data in Figs 5b and S5b to the following, Michaelis-Menten equation:

$$v_0([S]) = \frac{k_{cat}[E_0][S]}{K_m+[S]} \qquad (1)$$

Figures 5b and S5b contain data the correlates the initial reaction rate, v_0, to the substrate concentration. The initial reaction rate for a given substrate concentration was found by extrapolating the slope of the linear region (0-1 hours) of substrate hydrolysis (Fig S5a). The slope is equal to the initial reaction rate, which is the maximum rate for that given set of conditions.

First-order Rate Law Parameters:

The first-order rate constants were determined by finding the initial reaction rate for a given set of reaction conditions, as shown in Fig S5a. The effective first order rate constant was found by the relationship: $k_{obs} = v_0/[substrate]$. In figures 2, 4, S3, S4 and S8, we show first-order rate law best fit curves to the data, but these lines are only the aid the viewer and are not the fits used to determine rate constants. The curves are fits to the following equation, and the parameters for each dataset can be found in table S3:

$$y = A\left(1 - e^{-kt}\right) + B \qquad (2)$$

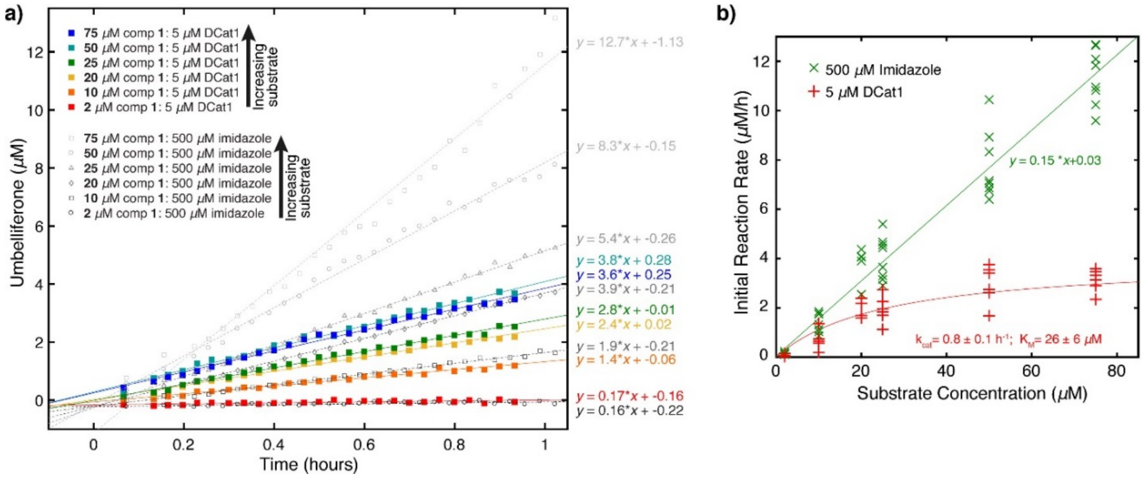

Figure S5: Analysis of DCat1 hydrolysis of substrate **1** to determine Michaelis-Menten parameters. a) Demonstration of how initial reaction rates were determined for one experiment repetition. The early time data (linear region) is fit to a line, allowing variation in the y-intercept due to background fluorescence. The slope is recorded as the initial reaction rate for each substrate level. b) the initial reaction rates found from n=4 (DCat 1) and n=6 (imidazole) experiments for both imidazole and DCat1 catalysts used to characterize the kinetics of each reaction.

Experiment	Parameter			Experiment	Parameter		
Figure 2c	A	B	k	***Figure 2d***	A	B	k
No Catalyst	1.17	0.08	0.06	*5 µM DCat1, n=9*	7.16	7.03	0.15
5 µM imidazole	0.86	0.06	0.10	*5 µM imidazole, n=11*	-73.21	-73.26	0.00
100 µM imidazole	3.81	0.06	0.08	*500 µM imidazole, n=11*	5.91	5.89	0.20
500 µM imidazole	5.42	0.08	0.22				
1 mM imidazole	6.05	0.09	0.39				
5 µM DCat1	6.46	0.13	0.18				
5 µM DCat2	4.22	0.09	0.09				
5 µM DCat4	2.91	0.08	0.08				
5 µM DCat7	3.08	0.09	0.10				
5 µM DCat10	5.00	0.12	0.15				
Figure 4c	A	B	k	***Figure 4d***	A	B	k
DCat1 + 1	7.07	6.66	0.09	*DCat1 + 1*	7.07	6.66	0.09
imidazole + 1	9.35	9.14	0.01	*imidazole + 1*	9.35	9.14	0.01
100x imidazole + 1	9.36	9.01	0.11	*100x imidazole + 1*	9.36	9.01	0.11
DCat1 + 2	10.67	10.40	0.01	*Dcat1+ 3*	13.09	11.37	0.01
imidazole + 2	4.46	4.19	0.04	*imidazole + 3*	4.53	3.06	0.03
100x imidazole + 2	7.67	7.88	0.42	*100x imidazole +3*	6.65	5.01	0.19
Figure S3	A	B	k				
5 µM DCat1 (red)	7.48	0.29	0.16				
5 µM oligo1+DSG adduct (gold)	16.67	0.18	0.01				
5 µM DSG (green)	14.08	0.17	0.00				
5 µM oligo1 (blue)	7.83	0.20	0.01				
Figure S8a	A	B	k	***Figure S8b***	A	B	k
DCat 1, n=2	6.25	0.18	0.12	*DCat7, n=2*	3.57	0.10	0.05
DCat 1 + random DNA n=2	6.07	0.21	0.13	*DCat7 + comp1 + rescue1, n=4*	3.35	0.10	0.06
DCat 1 + comp1 n=4	5.22	0.11	0.01	*DCat7 + comp1, n=4*	3.55	0.10	0.05
DCat 1 + comp1 + rescue1, n=4	5.90	-0.16	0.11	*5 µM imidazole, n=11*	4.37	0.05	0.02
DCat 1 + comp1 + rescue1, n=2	5.92	-0.80	0.10	*500 µM imidazole, n=11*	5.62	-0.09	0.22

Table S3: Observed first order fit parameters for figures 2, 4, S3, and S8

*Kinetics of imidazole-catalyzed hydrolysis of substrate **1**:*

To establish that imidazole-catalyzed hydrolysis of substrate **1** follows second-order kinetics, we checked whether reaction rate increases linearly with both substrate and catalyst (Fig S6), as is indeed the case. If the reaction follows the second order rate law, $v = k[catalyst][substrate]$, the two experiments shown in fig. S6 should appear as pseudo first-order, where the slope of the line of best fit in fig S6a is equal to $m = k[substrate\ \mathbf{1}] = 0.0022\ \mu M h^{-1}$ and the slope in fig S6b is equal to $m = k[imidazole] = 0.15\ \mu M h^{-1}$. That is, the experiment in fig S6a gives $k = 0.0022\ \mu M h^{-1}\ /10\ \mu M = 2.2{\times}10^{-4} h^{-1}$ while fig S6b gives $k = 0.15\ \mu M h^{-1}/500\ \mu M = 3.0{\times}10^{-4} h^{-1}$; these values are not significantly different. Therefore, we conclude that the imidazole-catalyzed hydrolysis of substrate **1** follows: $v = 3{\times}10^{-4} h^{-1}[imidazole][substrate\ \mathbf{1}]$.

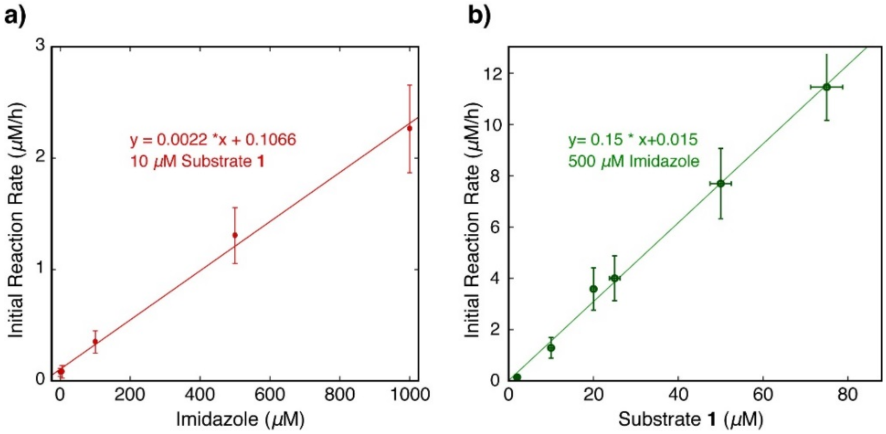

Figure S6: The imidazole-catalyzed hydrolysis of substrate **1** follows second order kinetics. a) The initial rate increases linearly with increasing concentration of imidazole catalyst. b) The initial rate increases linearly with increasing concentration of substrate **1**.

Uncatalyzed Hydrolysis Rates:

To account for background (uncatalyzed) hydrolysis of each ester substrate (**1**, **2**, **3**) under the reaction conditions, we determined k_{back}, the hydrolysis reaction rate constant for each substrate in the absence of imidazole and/or DCat (Figure S7). These reactions were monitored for 6 h. The slope of the line of best fit is the initial reaction rate and the rate constant is determined by dividing the initial reaction rate by the initial substrate concentration.

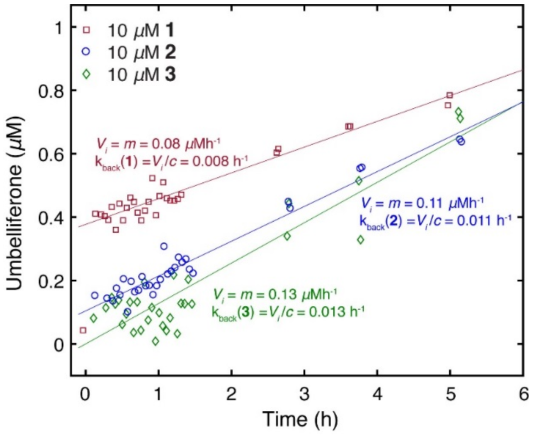

Figure S7: Determination of background hydrolysis rate constant, k_{back}, for each ester substrate.

Toehold-Switch Experiments:

DNA sequences used in Figure 3 are compiled in Table S4. The experiment shown in Figure 3 of the manuscript was also performed with **DCat7** as a control (Figure S8). Notably, **DCat7** was constructed with aptamer sequence **Ap2**, which has a different primary sequence than **Ap1**, which was used in **DCat1**. Therefore, **comp1** is not complementary to the DNA in **DCat7** (Table S1 and S4) and should not inhibit **DCat7** activity. Indeed, we see similar catalytic behavior of **DCat7** regardless of the inclusion of **comp1** or **rescue1**.

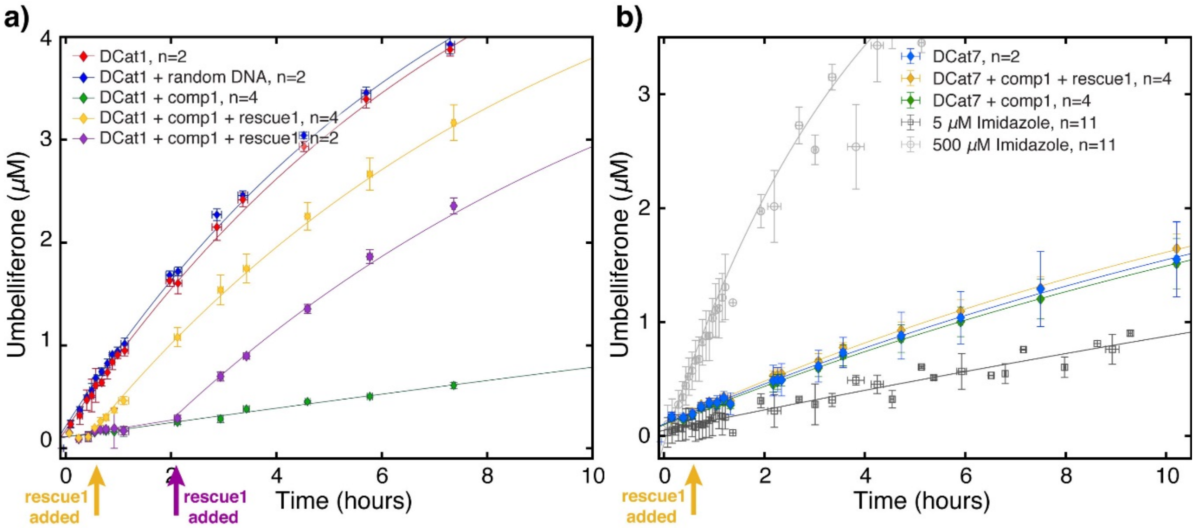

Figure S8: Control of DCat activity using toehold exchange shows catalytic behavior depends on aptamer structure: a) Experimental demonstration of toehold displacement rescue of DCat activity. The data shows product formation from hydrolysis of compound **1** with DCat1 under a series of conditions. DCat activity is suppressed by comp1 (green, yellow, purple diamonds) and then rescued at 35 minutes (yellow diamonds) or 130 minutes (purple diamonds) by the addition of rescue1 DNA, while "random" DNA does not suppress DCat activity (blue diamonds). Error bars represent 1 standard deviation, n as indicated. b) similar experiment with DCat7 in place of DCat1. Oligo rescue1, added at 35 minutes, does not change hydrolysis rate. Error bars represent 1 standard deviation, n as indicated.

oligo	DNA Sequence
comp1	5’ GCA GGA GAC TTG CGT CTG TCA ATT GAT CAT TAA TTC CAT TGA CCC TGC **GCG CAT GCA T** 3’
rescue1	5’ **A TGC ATG CGC** GCA GGG TCA ATG GAA TTA ATG ATC AAT TGA CAG ACG CAA GTC TCC TGC 3’
random	5’ AGC GCT TCT GCA TAC AGA ACG ATT TGG GTC AAT CGG CGC T’

Table S4: Sequences of oligonucleotides used in experiments in Fig 3 and Fig S8. Toehold is in bold.

Synthesis of esters:

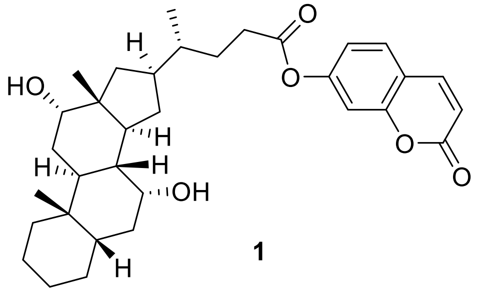

1

Under a nitrogen atmosphere, cholic acid (200 mg, 0.49 mmol, 1 equiv.), umbelliferone (0.49mmol, 79 mg, 1 equivalent), DMAP (12 mg, 0.10 mmol, 0.2 equiv.), and DCC (111 mg, 0.42 mmol, 1.1 equiv.) were added to a 10 mL round bottom flask. Anhydrous CH_2Cl_2 (2.5 mL, 39 mmol) was added and the resulting mixture was stirred overnight at room temperature. The precipitate was removed by celite filtration and concentrated. After column chromatography (2-4% MeOH gradient in CH_2Cl_2), **1** was isolated as a white solid (14 mg, 0.025 mmol, 5.2% yield). ^{1}H-NMR (500 MHz, $CDCl_3$): δ 7.72 (d, 1 H, *J* = 9.5 Hz), 7.51 (d, 1 H, *J* = 8.0 Hz), 7.13 (d, 1 H, *J* = 2.0 Hz), 7.07 (dd, 1 H, *J* = 8.5, 2.0 Hz), 6.42 (d, 1 H, *J* = 9.5 Hz), 4.03 (s, 1 H), 3.88 (s, 1 H), 3.49 (m, 1 H), 2.69 (m, 1 H), 2.56 (m, 1 H), 2.24 (m, 2 H), 2.01-1.01 (multiplet, 26 H), 0.92 (s, 3 H), 0.74 (s, 3 H). ^{13}C-NMR (125 MHz, $CDCl_3$): δ 12.6, 17.4, 22.5, 23.2, 26.7, 27.5, 28.4, 30.5, 30.7, 31.3, 34.6, 34.7, 35.1, 35.2, 39.6, 39.7, 41.4, 42.0, 46.5, 47.0, 68.4, 72.0, 72.9, 110.5, 116.0, 116.6, 118.4, 128.5, 142.9, 153.3, 154.7, 160.4, 172.0. HRMS (ESI) m/z: Calculated for $C_{33}H_{45}O_7^+$: 553.3160, found: 553.3161.

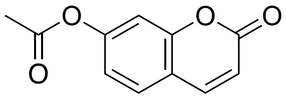

2

Under a nitrogen atmosphere, umbelliferone (195 mg, 1.2 mmol, 1 equiv) was added to 1.5 mL anhydrous diethylether in a 10 mL round bottom flask, the solution is cooled to 0°C and triethylamine (180 uL, 1.3 mmol, 1.1 equivalent) was added. A solution of acetyl chloride (100 µL, 1.4 mmol, 1.2 equivalents) in 1 mL diethyl ether was added dropwise and the reaction was stirred overnight. After column chromatography (9:1, hexanes/ethyl acetate eluent), **2** was isolated as a white solid. The spectral data were consistent with reported values.[4] ^{1}H-NMR (300 MHz, $CDCl_3$): δ 7.72 (d, 1 H, *J* = 8.7 Hz), 7.52 (d, 1 H, *J* = 7.5 Hz), 7.15 (d, 1 H, *J* = 2.1 Hz), 7.08 (dd, 1 H, *J* = 9.0, 2.1), 6.43 (d, 1 H, *J* = 9.9), 2.37 (s, 3 H)

Two-step synthesis of substrate **3**:

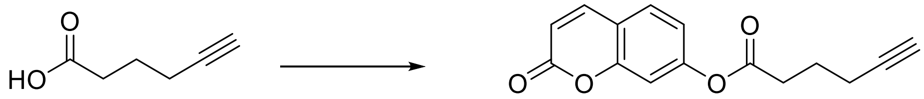

4

Figure S9: first step in synthesis of substrate **3**

To a 25 mL round bottom flask under N_2 equipped with magnetic stir bar was added CH_2Cl_2 (3.6 mL), 5-hexynoic acid (0.200 mL, 1.78 mmol, 1 equiv), EDC-HCl (0.376 g, 1.96 mmol, 1.1 equiv), and umbelliferone (0.376 g, 2.31 mmol, 1.3 equiv). Then DMAP (0.022 g, 0.18 mmol, 0.1 equiv) was added. The reaction mixture was stirred overnight at room temperature and the solvent was then removed *en vacuo*. After column chromatography (4:1 hexanes/ethyl acetate eluent), **4** was isolated as a white solid (235.5 mg, 52% yield), which was carried forward to the next step. ^{1}H NMR ($CDCl_3$, 500 MHz): δ 7.71 (d, 1 H, *J* = 9.5 Hz), 7.51 (d, 1 H, *J* = 8.5 Hz), 7.15 (d, 1H, *J* = 2), 7.08 (dd, 1 H, *J* = 8.5, 2.0 Hz), 6.42 (d, 1 H, *J* = 9.5 Hz), 2.79 (t, 2 H, *J* = 7.5 Hz), 2.39 (td, 2 H, *J* = 7.0, 3.0 Hz), 2.05 (t, 1 H, *J* = 2.7 Hz), 2.01 (quin, 2 H, *J* = 7.2 Hz). ^{13}C NMR ($CDCl_3$, 125 MHz) δ 17.8, 23.3, 32.9, 69.6, 82.8, 110.4, 116.1, 116.7, 118.4, 128.5, 142.8, 153.2, 154.7, 160.3, 170.9.

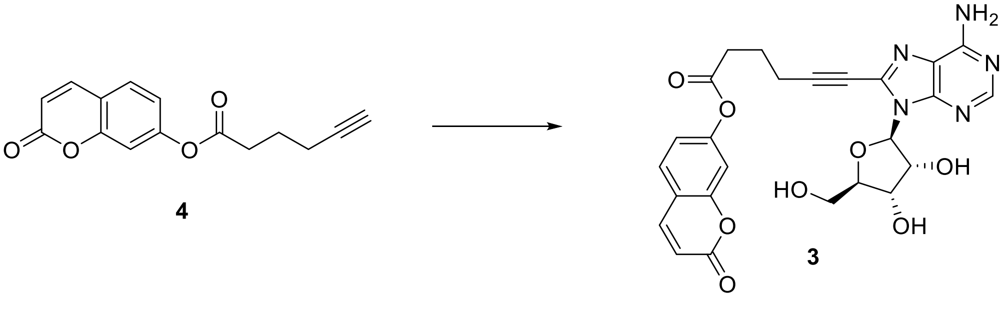

Figure S10: Final step in synthesis of substrate **3**.

This procedure was adapted from Lambertucci et al.[5] To a 25 mL round bottom flask under N_2 equipped with magnetic stir bar was added 8-bromoadenosine (0.050 g, 0.15 mmol, 1.0 equiv), DMF (4.8 mL), and Et_3N (0.65 mL, 4.60 mmol, 32 equiv). $PdCl_2(PPh_3)_2$ (0.005 g, 0.008 mmol, 0.05 equiv) and CuI (0.0003 g, 0.0016 mmol, 0.013 equiv) were added, followed by **4** (0.111 g, 0.043 mmol, 3.0 equiv). The reaction mixture was stirred at room temperature for 60 h, and the DMF was then removed by rotary evaporation. The adenosine ester (**3**, 15.6 mg, 20% yield) was obtained as a white solid after two rounds of column

chromatography (19:1 CH_2Cl_2/MeOH). ^{1}H-NMR (500 MHz, CD_3OD): δ 8.19 (s, 1 H), 7.95 (d, 1 H, *J* = 9.5 Hz), 7.65 (d, 1 H, *J* = 8.0), 7.25 (d, 1 H, *J* = 2 Hz), 7.17 (dd, 1 H, *J* = 8.5 Hz, 2 Hz), 6.42 (d, 1 H, *J* = 9.5 Hz), 6.19 (d, 1 H, *J* = 7.5 Hz), 5.03 (dd, 1 H, *J* = 7.0 Hz, 5.5 Hz), 4.38 (dd, 1 H, *J* = 4.9 Hz, 1.5 Hz), 4.21 (d, 1 H, *J* = 1.5 Hz), 3.90 (dd, 1 H, *J* = 12.5 Hz, 6.5 Hz), 3.74 (t, 1 H, *J* = 13.5 Hz), 2.91 (t, 2 H, *J* = 7.5 Hz), 2.79 (t, 2 H, *J* = 7.0 Hz), 2.16 (quin, 2 H, *J* = 7.0 Hz). ^{13}C-NMR (125 MHz, CD_3OD): δ 7.83, 17.99, 22.67, 32.42, 62.69, 69.83, 71.80, 72.84, 87.49, 90.49, 97.48, 109.87, 115.07, 116.76, 118.37, 128.75, 135.14, 143.64, 148.09, 152.57, 153.44, 154.44, 155.90, 160.94, 171.03. HRMS (ESI) m/z: Calculated for $C_{25}H_{24}N_5O_8^+$: 522.1619, found: 522.1628.

NMR data:

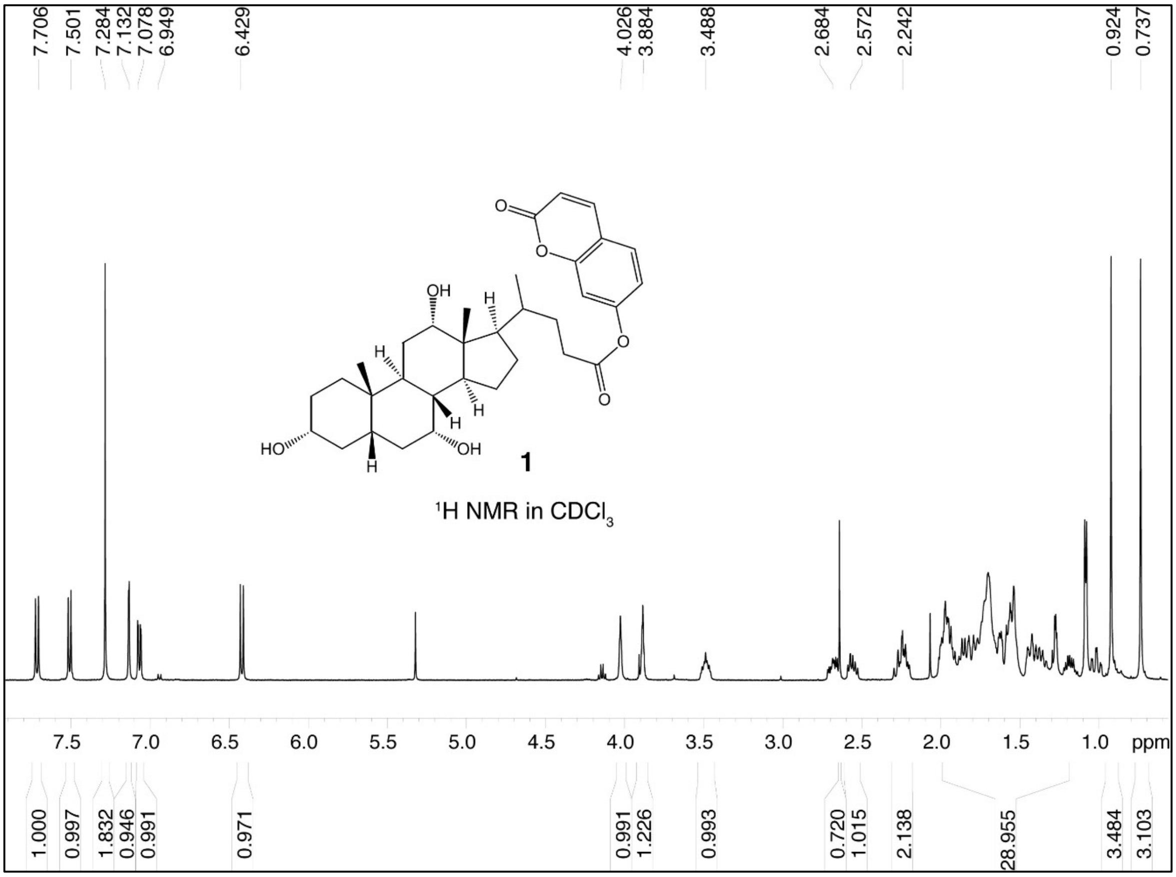

7.706
7.501
7.284
7.132
7.078
6.949
6.429
4.026
3.884
3.488
2.684
2.572
2.242
0.924
0.737
OH
H
HO
OH
O
1
¹H NMR in CDCl₃
7.5 7.0 6.5 6.0 5.5 5.0 4.5 4.0 3.5 3.0 2.5 2.0 1.5 1.0 ppm
1.000
0.997
1.832
0.946
0.991
0.971
0.991
1.226
0.993
0.720
1.015
2.138
28.955
3.484
3.103

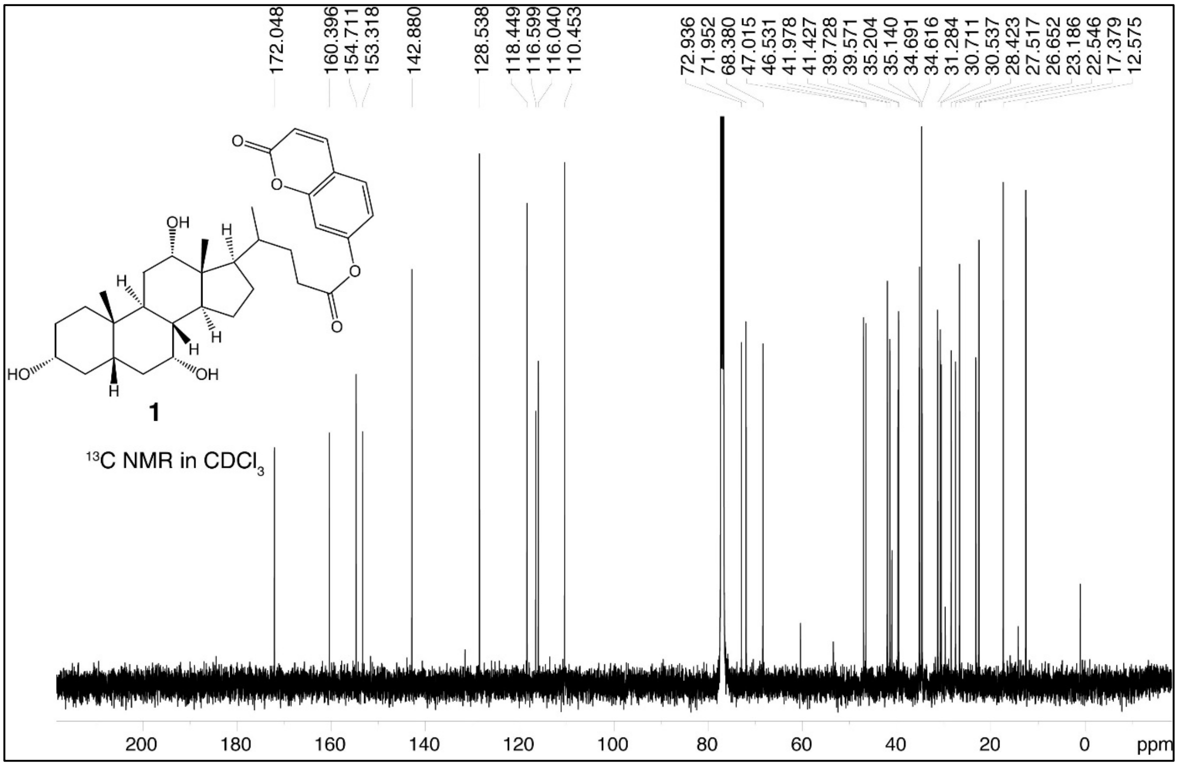

172.048
160.396
154.711
153.318
142.880
128.538
118.449
116.599
116.040
110.453
72.936
71.952
68.380
47.015
46.531
41.978
41.427
39.728
39.571
35.204
35.140
34.691
34.616
31.284
30.711
30.537
28.423
27.517
26.652
23.186
22.546
17.379
12.575
OH
H
HO
OH
O
1
¹³C NMR in CDCl₃
200 180 160 140 120 100 80 60 40 20 0 ppm

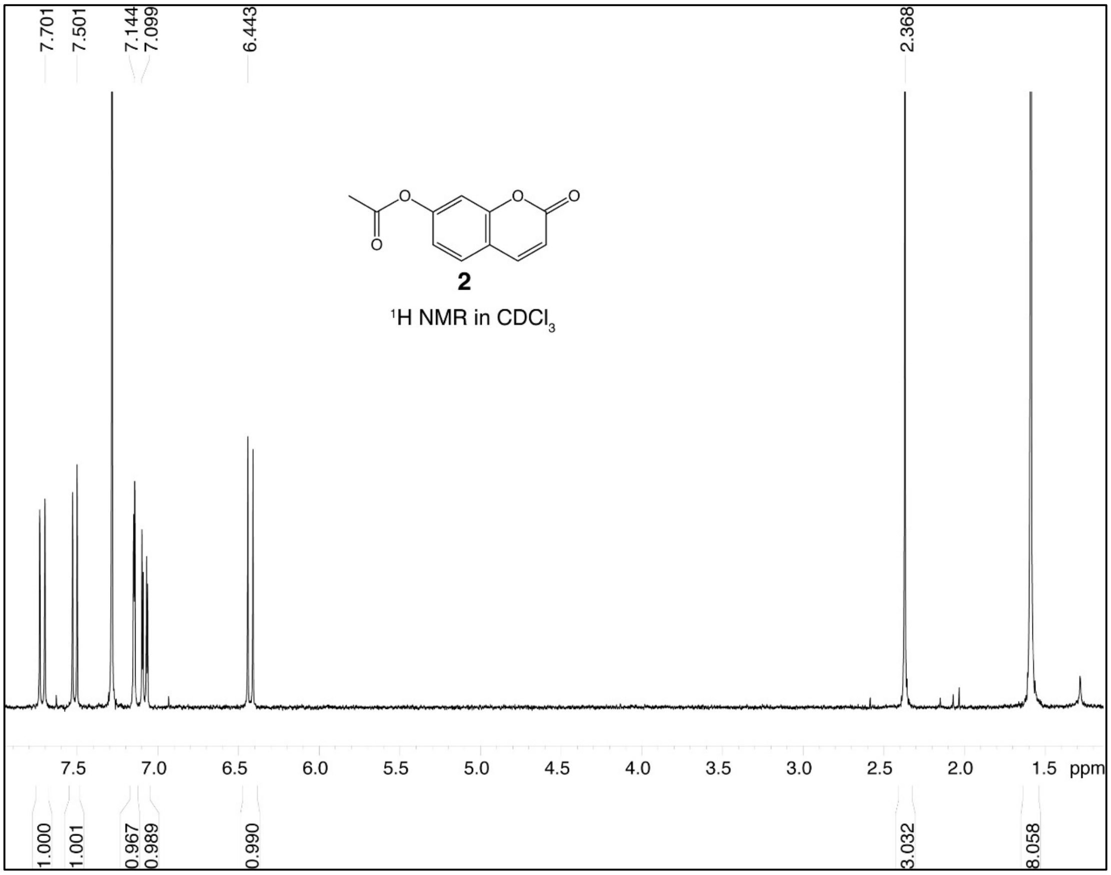
7.701
7.501
7.144
7.099
6.443
2.368
O
O
O
O
2
¹H NMR in CDCl₃
7.5 7.0 6.5 6.0 5.5 5.0 4.5 4.0 3.5 3.0 2.5 2.0 1.5 ppm
1.000
1.001
0.967
0.989
0.990
3.032
8.058

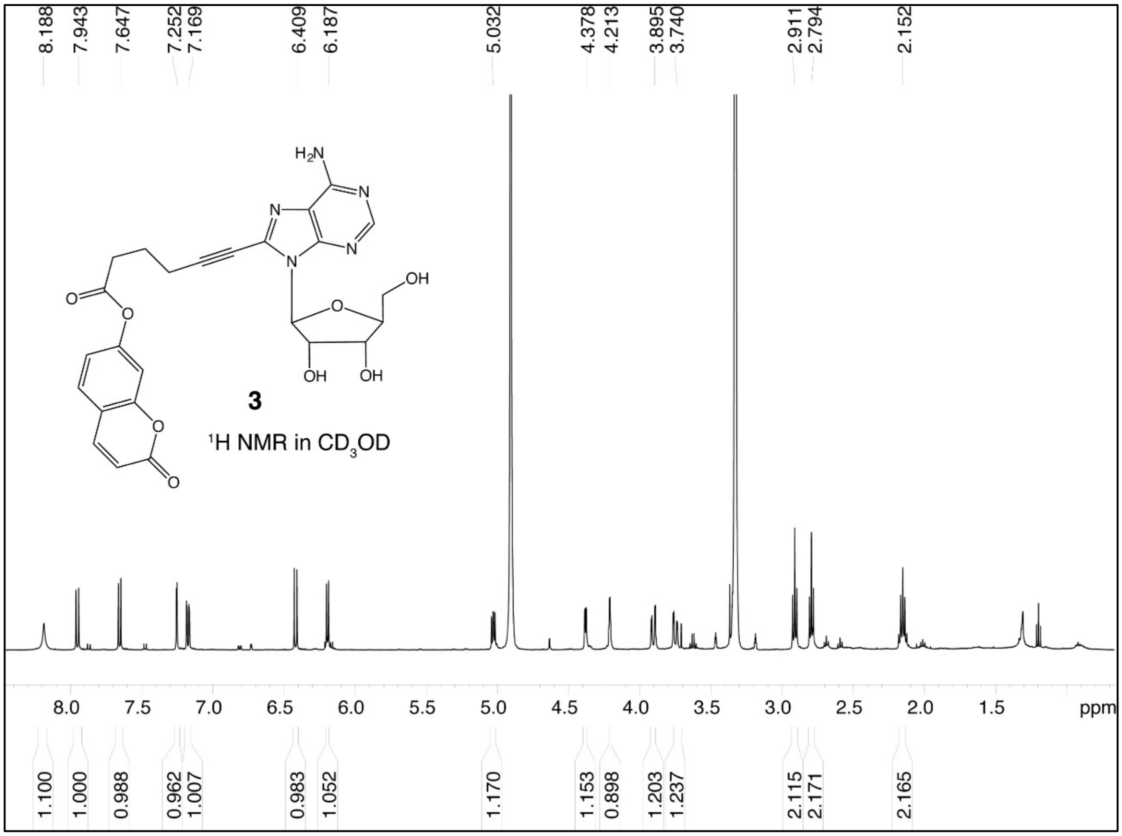
8.188
7.943
7.647
7.252
7.169
6.409
6.187
5.032
4.378
4.213
3.895
3.740
2.911
2.794
2.152
H₂N
N
N
N
N
OH
O
OH
OH
O
O
O
O
O
3
¹H NMR in CD₃OD
8.0 7.5 7.0 6.5 6.0 5.5 5.0 4.5 4.0 3.5 3.0 2.5 2.0 1.5 ppm
1.100
1.000
0.988
0.962
1.007
0.983
1.052
1.170
1.153
0.898
1.203
1.237
2.115
2.171
2.165

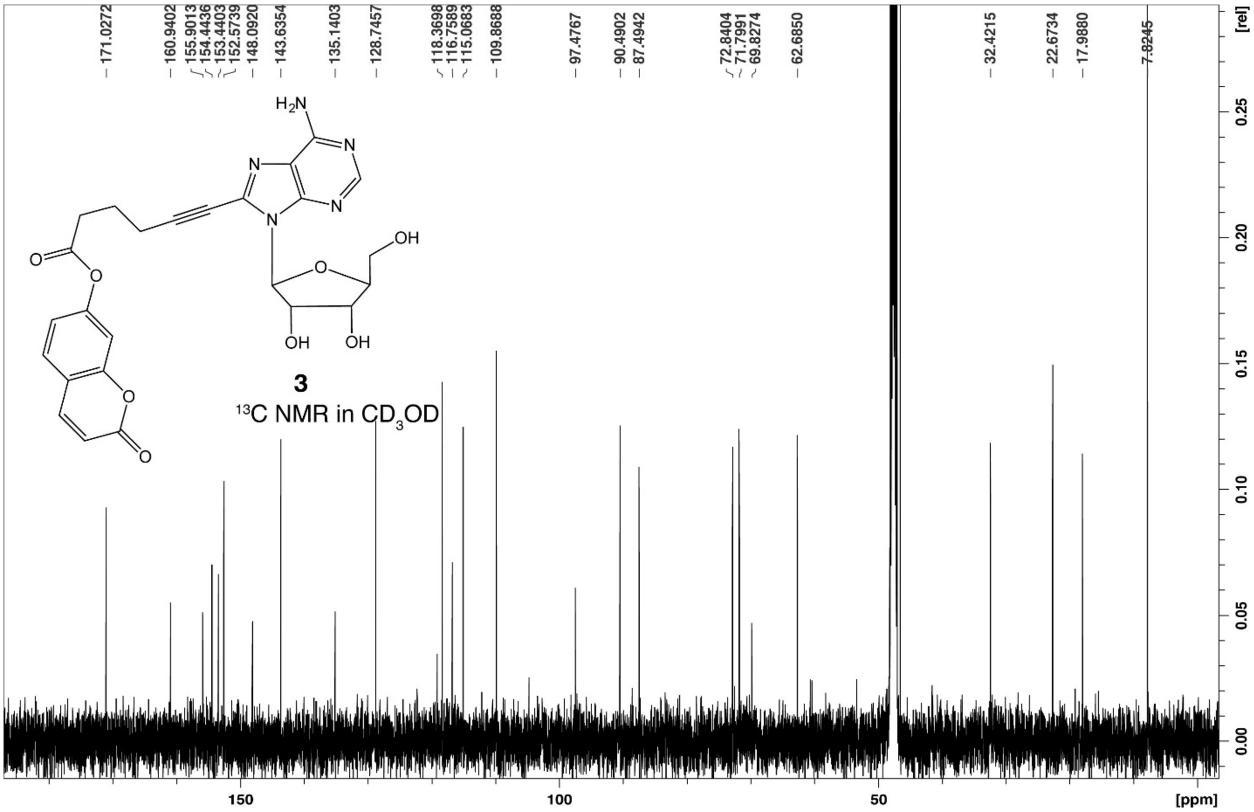
171.0272
160.9402
155.9013
154.4436
153.4403
152.5739
148.0920
143.6354
135.1403
128.7457
118.3698
116.7589
115.0683
109.8688
97.4767
90.4902
87.4942
72.8404
71.7991
69.8274
62.6850
32.4215
22.6734
17.9880
7.8245
[rel]
0.25
0.20
0.15
0.10
0.05
0.00
150
100
50
[ppm]
H_2N
N
O
OH
OH
OH
3
^{13}C NMR in CD_3OD

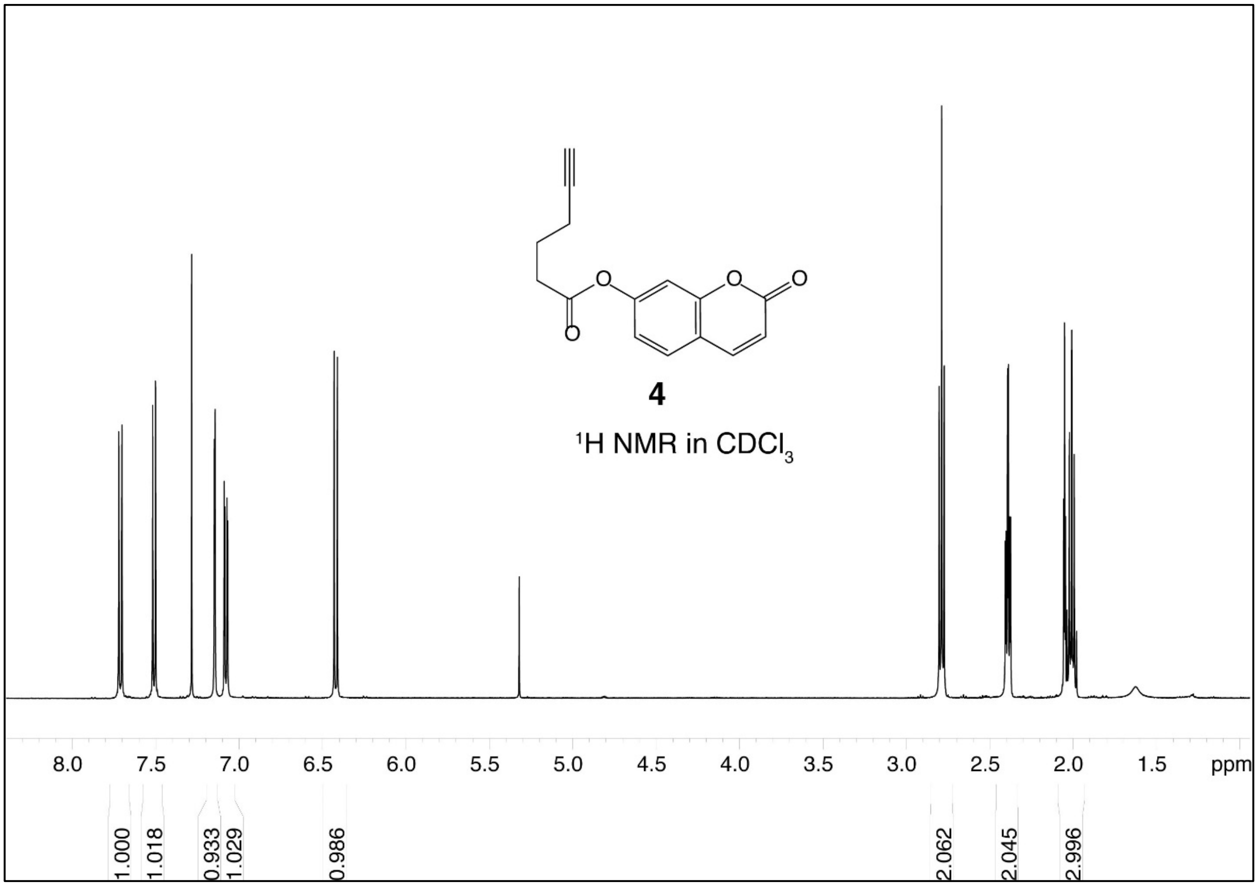
O
O
O
O
4
^1H NMR in $CDCl_3$
8.0
7.5
7.0
6.5
6.0
5.5
5.0
4.5
4.0
3.5
3.0
2.5
2.0
1.5
ppm
1.000
1.018
0.933
1.029
0.986
2.062
2.045
2.996

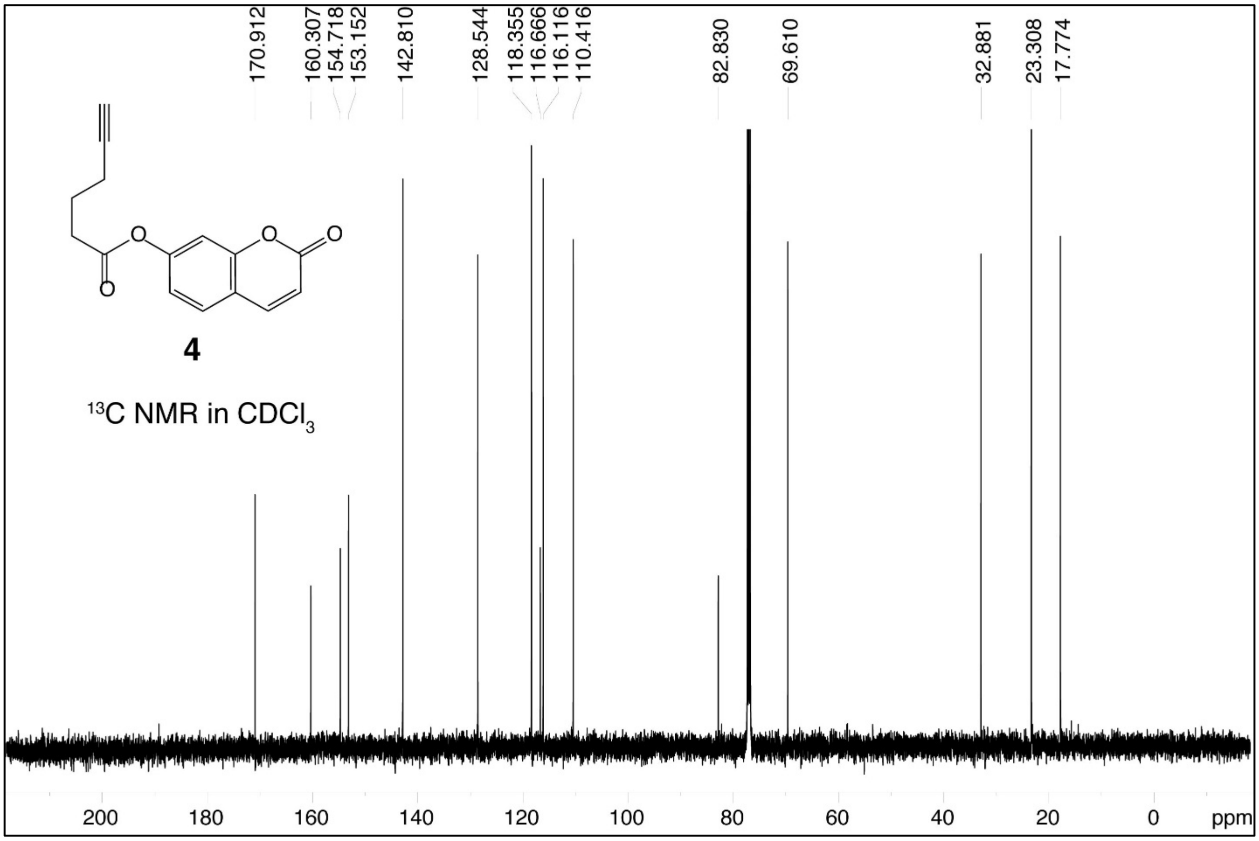
170.912
160.307
154.718
153.152
142.810
128.544
118.355
116.666
116.116
110.416
82.830
69.610
32.881
23.308
17.774
4
^{13}C NMR in $CDCl_3$
200
180
160
140
120
100
80
60
40
20
0
ppm

References:

1 T. Kato, K. Yano, K. Ikebukuro and I. Karube, *Nucleic Acids Res.*, 2000, **28**, 1963–1968.

2 T. Kato, T. Takemura, K. Yano, K. Ikebukuro and I. Karube, *Biochim. Biophys. Acta BBA - Gene Struct. Expr.*, 2000, **1493**, 12–18.

3 DCats are heated at 95 °C to melt all hybridization and tertiary structure, followed by a slow cool to 37 °C to encourage proper DNA folding and tertiary structure. For example: B. Högberg, T. Liedl and W. M. Shih, *J. Am. Chem. Soc.*, 2009, **131**, 9154–9155.

4 W. He, B.-L. Zhang, S.-Y. Zhou, X.-L. Sun and S.-Y. Zhang, *Synth. Commun.*, 2007, **37**, 361–367.

5 C. Lambertucci, S. Costanzi, S. Vittori, R. Volpini and G. Cristalli, *Nucleosides Nucleotides Nucleic Acids*, 2001, **20**, 1153–1157.
